# Alterations in the hepatic drug-metabolizing enzyme network of mice induced by +Gz exposure and its impact on the pharmacokinetics and pharmacodynamics of modafinil

**DOI:** 10.3389/fphar.2026.1818737

**Published:** 2026-08-03

**Authors:** Fengzhou Liu, Hui Shen, Qun Wan, Yajuan Li, Gang Zhao, Xiao Liu, Wei Lin, Junhui Xue

**Affiliations:** 1 Aerospace Clinical Medicine Center, Air Force Medical University, Xi’An, China; 2 Aviation Medicine Department, First Affiliated Hospital of Air Force Medical University, Xi’An, China; 3 Innovation Research Institute, Xijing Hospital, Air Force MedicalUniversity, Xi’an, China

**Keywords:** +Gz, aviation pharmacology, drug metabolism, liver injury, modafinil, pharmacokinetics

## Abstract

**Background:**

In aviation and aerospace missions, personnel and astronauts are subjected to brief episodes of +Gz acceleration, resulting in overload stress that adversely affects the cardiovascular, respiratory, and nervous systems. This mechanical stress disrupts hemodynamic balance, tissue oxygenation, and physiological homeostasis. Increasing evidence links this stressor to hepatic injury; as the primary organ for drug metabolism, liver dysfunction may alter the expression and activity of drug-metabolizing enzymes and the pharmacokinetic profiles of circadian rhythm-regulating medications, such as modafinil. This study systematically evaluates the effects of repeated brief + Gz exposure on hepatic drug-metabolizing enzymes and modafinil pharmacokinetics using a murine model.

**Methods:**

A mouse model was established using a 7-day continuous +15 Gz exposure regimen. The anti-fatigue effect of modafinil under + Gz and sleep deprivation conditions was assessed using the Morris water maze. Modafinil plasma concentration was analyzed by ultra-high-performance liquid chromatography (UHPLC) to determine pharmacokinetic parameters. Transcriptomic sequencing, RT-qPCR, Western blot, and enzyme activity assays were employed to comprehensively evaluate the expression of hepatic drug metabolism-related genes and the expression/activity of the key enzyme Cyp3a11.

**Results:**

+Gz exposure impaired spatial learning and memory in mice, showing a synergistic negative effect with 24-h sleep deprivation. Modafinil intervention significantly ameliorated these cognitive deficits, indicating retained anti-fatigue efficacy under acceleration stress. +Gz exposure induced mild hepatic dysfunction (elevated ALT/AST). Pharmacokinetic analysis revealed a reduced peak plasma concentration (C_max_), along with significantly prolonged mean residence time (MRT) and elimination half-life (t_1/2z_) of modafinil in the +Gz group, characterizing a pattern of “delayed absorption-impaired clearance”. Transcriptomic analysis showed that + Gz exposure significantly downregulated the transporter Slc17a9 and Ugt2b37, while upregulating Slc25a34/Slc25a47, Abcg5/Abcg8, and the metabolizing enzymes Cyp2b10 and Cyp2c38. Modafinil partially reversed these alterations. The key finding was that + Gz exposure did not alter the mRNA or protein expression levels of Cyp3a11, the principal enzyme responsible for modafinil metabolism, but significantly suppressed its enzymatic activity.

**Conclusion:**

Brief repeated + Gz stress reprograms the hepatic drug-metabolizing gene expression profile, potentially affecting the metabolism of various drugs in the unique conditions of aerospace environments. Importantly, it functionally inhibits the activity of the key metabolic enzyme Cyp3a11, without altering its expression, resulting in characteristic changes in modafinil pharmacokinetics.

## 1 Introduction

In aviation and spaceflight activities, flight personnel and astronauts are subjected to high acceleration (+Gz) during specific periods, such as in the steep climbs of fighter jets or during spacecraft launches. These conditions exert substantial impacts on the cardiovascular and central nervous systems while inducing either adaptive or damaging changes in the function of multiple organs (Shi et al., 2019). Among these effects, the influence of sustained high + Gz environments on liver function has garnered increasing attention. Previous studies have shown that prolonged exposure to + Gz triggers disordered hepatocellular structure, elevated oxidative stress levels, and abnormal liver function indicators in experimental animals, such as mice, providing clear evidence of hepatic injury (Shi et al., 2015). Given its role as a central hub for drug metabolism, the liver’s functional status directly regulates the expression and activity of drug-metabolizing enzymes, thereby modulating the pharmacokinetic processes of medications (Rodrigues et al., 2022).

Modafinil, as a circadian rhythm-regulating drug, helps maintain alertness during tasks and counteracts daytime sleepiness caused by sleep deprivation and circadian rhythm disruptions (Ooi et al., 2019). It has been widely utilized in aviation and aerospace activities for fatigue management among flight personnel and astronauts during mission execution (Hersey and Tanda, 2024; Repantis et al., 2021). Its metabolism mainly relies on the liver CYP enzyme system, especially subtypes such as CYP3A4 (Yuan et al., 2020). However, there is no clear research on whether the liver drug-metabolizing enzymes undergo functional changes under the special environmental factors of aviation (especially continuous + Gz), which in turn affect the metabolism process of modafinil. Our previous preliminary experimental results indicate that continuous + Gz exposure may lead to significant changes in the expression and activity of various drug-metabolizing enzymes in the liver of mice, suggesting that this environmental factor may affect the pharmacological effect and toxicological response of the drug by regulating the metabolic enzyme system.

Therefore, this study aims to systematically investigate the effects of continuous + Gz exposure on the expression and function of drug-metabolizing enzymes in the liver of mice, and further analyze its impact on the pharmacokinetics of modafinil. This will provide experimental evidence for rational drug use in aviation environments and flight health assurance.

## 2 Materials and methods

### 2.1 Animals

Adult male C57BL/6J mice (8–10 weeks old) were housed under standard conditions and randomly assigned to various experimental groups as detailed below. All experimental procedures were approved by the Institutional Animal Care and Use Committee of Fourth Military Medical University (Xi’an, China).

### 2.2 Materials

Modafinil (H20170013) and Placebo were purchased from Welman PHARMACEUTICAL (Hubei China). The antibody of CYP3A4 (Cyp3a11 isoform in mice) was gained from Abcam (ab3572). Methanol and acetonitrile were using HPLC grade, available from (China). Formic acid was chromatographed and purchased from (China).

### 2.3 Behavioral assessment

#### 2.3.1 Experimental design and pharmacological intervention

##### 2.3.1.1 Experimental design

Male C57BL/6J mice (8–10 weeks old) were subjected to + Gz exposure for 7 consecutive days. The Morris Water Maze (MWM) training protocol commenced on the 3rd day of +Gz exposure. Following the navigation training on the 4th day, mice were subjected to 24 h of sleep deprivation (SD). On the 7th day, immediately following the final + Gz exposure, mice were administered Modafinil (32 mg/kg) via oral gavage. The spatial probe trial was conducted 1 h post-administration.

##### 2.3.1.2 Animal grouping and screening

To ensure baseline cognitive homogeneity, all mice underwent a preliminary MWM screening. Individuals exhibiting exceptionally high learning capabilities were excluded. The remaining mice were randomly assigned into four groups (n = 8 per group, total n = 32): (1) Control group; (2) +Gz group; (3) +Gz + SD24 h group; and (4) +Gz + SD24 h + Modafinil group.

##### 2.3.1.3 Pharmacological intervention

The dosage of Modafinil (32 mg/kg) for the MWM test was determined based on previous literature regarding the pharmacodynamics and pharmacokinetics of Modafinil under + Gz exposure conditions (Cope et al., 2017; Fernandes et al., 2013; Fernandes et al., 2015; Béracochéa et al., 2008; Morgan et al., 2007).

#### 2.3.2 Morris water maze (MWM) test

The MWM test was utilized to evaluate spatial learning and memory. The place navigation test lasted for four consecutive days. A hidden platform was positioned in the first quadrant. Mice underwent four training trials per day with an inter-trial interval exceeding 1 h. The starting quadrant for the first trial of Day 1 was the first quadrant, followed sequentially by the second, third, and fourth quadrants. On Day 2, the starting quadrant began from the second quadrant, continuing sequentially thereafter. The time required for a mouse to locate the platform within 60 s was recorded as the escape latency. If a mouse successfully located the platform, it was allowed to remain on it for 5 s before being retrieved, dried, and returned to its home cage. If a mouse failed to find the platform within 60 s, the escape latency was recorded as 60s, and the mouse was gently guided onto the platform and allowed to stay for 15 s.

One hour prior to the formal spatial probe test on the final day, mice received an intragastric administration of the drug or vehicle. For the probe test, the platform was removed, and each mouse was placed in the opposite third quadrant. The time to the first crossing of the former platform location, the number of platform crossings, and the time spent in the target quadrant during the 60 s trial were recorded for statistical analysis.

#### 2.3.3 +Gz exposure protocol

Based on previous studies and our preliminary data, a +Gz acceleration of 15 G was selected. Mice were placed in a customized restraint device mounted on a small-animal centrifuge. The exposure was conducted once daily for 15 min over 7 consecutive days. Following each session, mice were returned to their home cages.

#### 2.3.4 Modified multiple platform method for sleep deprivation

Sleep deprivation (SD) was induced using the modified multiple platform method. The apparatus consisted of a water tank containing multiple platforms (diameter: 2.5 cm) positioned 1 cm above the water surface. Mice could stand on the platforms or engage in non-rapid eye movement (NREM) sleep. However, upon the onset of rapid eye movement (REM) sleep, the resulting muscle atonia caused the mice to touch the water, thereby preventing the completion of the sleep cycle. The duration of SD was strictly maintained at 24 h.

### 2.4 Pharmacokinetic study of modafinil

#### 2.4.1 Drug administration and blood sample collection

Mice from the pharmacokinetic study groups received modafinil (120 mg/kg) (Fernandes et al., 2013; Fernandes et al., 2015). Blood samples (0.3 mL) were collected via the orbital plexus at 0.25, 0.5, 1, 2, 4, 6, and 8 h post-administration. Plasma was separated and stored at −80 °C until analysis.

#### 2.4.2 Chromatographic conditions

The Waters Acquity ARC HPLC system (Waters, United States of America) was utilized for HPLC analysis. A Symmetry® C18 column (5 μm, 4.6 mm × 250 mm) was employed, with the column temperature maintained at 30 °C. The mobile phase is made up of ultra-pure water and chromatography-grade methanol in a 45:55 volume-to-volume ratio, with a flow rate of 0.25 mL per minute. The detection wavelength is set at 220 nm, and the sample injection volume is 3 µL.

#### 2.4.3 Pretreatment of plasma samples

A 100 μL aliquot of plasma was placed into a 1.5 mL Eppendorf tube, and 250 μL of acetonitrile (2.5 times the volume of plasma) was added. The resulting mixture was vortexed vigorously for 1 min and then centrifuged at 8000 rpm for 15 min at 4 °C. The supernatant was subsequently collected for HPLC analysis.

#### 2.4.4 Construction of the standard curve

A stock solution of modafinil was prepared at a concentration of 1 mg/mL and subsequently serially diluted with blank plasma to create calibration standards at concentrations of 0.5, 1.0, 5.0, 10, 20, 50, 80, and 100 mg/L. For each of these standards, acetonitrile was added at a volume 2.5 times that of the sample. The mixtures were thoroughly vortexed and then centrifuged at 8000 rpm for 15 min at 4 °C. The supernatants were collected for HPLC analysis. A standard curve was generated through linear regression analysis of the modafinil peak areas against the corresponding nominal concentrations.

#### 2.4.5 Methodological validation

In this study, we established a comprehensive method for the analysis of modafinil in plasma samples obtained from male C57BL/6J mice, utilizing HPLC. For specificity, blank plasma was processed alongside modafinil standards, and chromatograms were compared to identify modafinil’s retention time and peak characteristics, ensuring no interfering peaks were present. The precision and accuracy of the method were assessed by spiking 10 μL of standard solution at concentrations of 1, 10, and 50 mg/L into 90 μL of plasma, followed by acetonitrile addition and subsequent centrifugation. The samples were evaluated within the same day and subsequently stored to assess stability over several days, with accuracy calculated as the ratio of measured to theoretical values multiplied by 100%, and precision expressed as relative standard deviation (RSD). Recovery was determined by comparing the peak areas of modafinil in spiked plasma samples to those obtained from processed blank plasma. To evaluate stability, samples were assessed after different storage periods-0h, 4 h at room temperature, 24 h at 4 °C, 10 days at −20 °C, and 1 month at −80 °C following the same processing protocol, with the supernatant analyzed to ensure reliable quantification of modafinil over time.

#### 2.4.6 Pharmacokinetic parameter calculation

Using the pharmacokinetic software DAS 2.0, we performed compartmental modeling of the plasma drug concentration of modafinil in mice that were administered the drug via gavage. Following oral gavage administration of modafinil to mice, the peak plasma concentration (C_max_) and the time to reach Cmax (T_max_) were determined directly from the observed data. The elimination rate constant (K) was estimated by linear regression of the terminal phase of the concentration-time curve. The elimination half-life (t_1/2_) was calculated as t_1/2_ = 0.693/K. The area under the concentration-time curve (AUC) was derived using the trapezoidal method, and the mean residence time (MRT) was calculated as MRT = AUMC/AUC.

### 2.5 Transcriptome sequencing and bioinformatic analysis

#### 2.5.1 RNA high-throughput sequencing

##### 2.5.1.1 Sample preparation and library construction

Total RNA was extracted from liver tissues of both the experimental and control group mice using Trizol reagent, following the manufacturer’s instructions. The quality and integrity of the extracted RNA were assessed. cDNA libraries were then constructed using a total RNA-seq library preparation kit according to the standard protocol. The quality of the final cDNA libraries was evaluated prior to sequencing.

##### 2.5.1.2 Sequencing

The cDNA libraries were subjected to high-throughput sequencing on an Illumina HiSeq platform (Illumina, San Diego, CA, United States of America), generating paired-end reads. The transcriptome sequencing service was provided by Tsingke Biotechnology Co., Ltd (Beijing, China).

#### 2.5.2 Gene expression analysis

##### 2.5.2.1 Screening of differentially expressed genes (DEGs)

Differential gene expression analysis between the experimental and control groups was performed. Genes with an absolute Fold Change (FC) ≥ 2 and an adjusted p-value < 0.05 were considered statistically significant DEGs. The results of the DEG analysis were visualized using a volcano plot and a hierarchical clustering heatmap.

##### 2.5.2.2 Go and KEGG annotation and pathway enrichment analysis

To elucidate the biological functions and pathways associated with the identified DEGs, Gene Ontology (GO) and Kyoto Encyclopedia of Genes and Genomes (KEGG) pathway annotation and enrichment analysis were conducted. The DEGs were mapped to the GO and KEGG database to retrieve pathway annotations. Enrichment analysis was then performed, and significantly enriched pathways were categorized based on the GO and KEGG pathway classification system.

### 2.6 Real-time PCR method

Total RNA was extracted from liver tissues, reverse-transcribed into cDNA, and analyzed by quantitative real-time PCR using the primers listed in Table 1. Gene expression was normalized to β-actin.

**TABLE 1:** Primer sequences used for real-time PCR.

Gene	Gene ID	Forward primer (5′-3′)	Reverse primer (5′-3′)
β-actin	11,461	AAC​AGT​CCG​CCT​AGA​AGC​AC	CGT​TGA​CAT​CCG​TAA​AGA​CC
Abcg5	27,409	TGC​CAT​CCT​GAC​TTA​CGG​AGA​G	CTG​CTT​TGG​GTG​TCC​ACT​GAT​G
Abcg8	67,470	GGT​CCT​TCT​GAT​GAC​ATC​TGG​C	CGT​CTG​TCG​ATG​CTG​GTC​AAG​T
Ugt2b37	112,417	GTG​CTC​AAT​GCA​CTG​GAG​GAA​G	CTC​AAC​CCA​GAA​TAC​AGC​CCT​G
Slc17a9	228,993	TTG​GCT​GGA​ACA​AGA​AGG​AGG​C	GAC​AGC​AAG​ATG​ACC​TTC​TCT​CC
Slc25a47	104,910	CTC​AGG​ATG​TGC​CTC​TGG​TCT​T	AAG​CAG​TGG​GTG​TGG​GAC​ACA​A
Slc25a34	384,071	CAG​TAT​AGC​CGT​GGT​TGC​AGT​C	AGG​TCT​TCA​CCA​GGC​AAT​CAG​C
Cyp2c38	13,097	CCT​TGT​CCC​TAA​CAA​CCT​ACC​C	GGA​ACT​CCT​TGC​TGT​CAT​GCA​G
Cyp2b10	13,088	TGC​TGT​CGT​TGA​GCC​AAC​C	CCA​CTA​AAC​ATT​GGG​CTT​CCT

### 2.7 Measurement of Cyp3a11 activity

Modafinil is metabolized by Cyp3a11 of cytochrome P450 system in liver to produce two major metabolites, modafinil acid and modafinil sulfone. Protein from liver tissues were isolated by centrifugation. The livers were removed and washed immediately with PBS. The homogenate was centrifuged at 3000rpm for 20 min, and the supernatant was collected. The activity of Cyp3a11 was determined by ELISA kit (MEIMIAN, CHINA). The Cyp3a11 activity was determined based on a standard curve under the assay conditions.

### 2.8 Western blot

Following sacrifice, liver tissues were promptly excised and homogenized in an appropriate buffer. The homogenate was centrifuged at 12,000 × g for 20 min at 4 °C, and the resulting supernatant was collected. Total protein concentration was determined using a BCA protein assay kit according to the manufacturer’s instructions. Equal amounts of protein (30–50 μg) were separated by SDS-polyacrylamide gel electrophoresis and subsequently transferred onto PVDF membranes. The membranes were blocked with 5% BSA for 2 h at room temperature and then incubated overnight at 4 °C with a primary antibody against Cyp3a11 at a dilution of 1:1,000. After incubation, the membranes were washed three times with TBST and incubated for 2 h at room temperature with a horseradish peroxidase-conjugated secondary antibody diluted at 1:5,000 in TBST. Protein bands were visualized using an ECL Plus western blotting detection kit and exposed to photographic film. The resulting bands were scanned and quantified using a chemiluminescence imaging system.

### 2.9 Statistical analysis

Transcriptome raw counts were normalized using DESeq2 v1.32.0. Differentially expressed genes were identified with |log2FC| ≥ 1 and adjusted *P* < 0.05 (Benjamini–Hochberg correction). Principal component analysis (PCA) was performed to assess batch effects, and three biological replicates were used per group.

Data are presented and compared as the means. The software package SPSS19.0 and DAS2.0 software was used for the analysis. Two-way analysis of variance was used, followed by Dunnett’s test. The test was performed when the variances were equal, and the test was performed when the variances were not equal. The results were considered statistically significant when the probability was *P* < 0.05.

## 3 Results

### 3.1 The effect of modafinil on the learning and memory abilities of sleep-deprived mice exposed to +Gz

#### 3.1.1 Exposure to +Gz leads to a decline in the learning and memory abilities of mice

Between 8 and 10 weeks of age, adult male C57BL/6J mice underwent a daily +15 Gz acceleration paradigm, with each session lasting 15 min over 7 consecutive days. The Morris water maze test was initiated 1 hour after the acceleration intervention on the third day of exposure. A spatial probe trial was conducted on the fifth day of the water maze testing.

**FIGURE 1:**
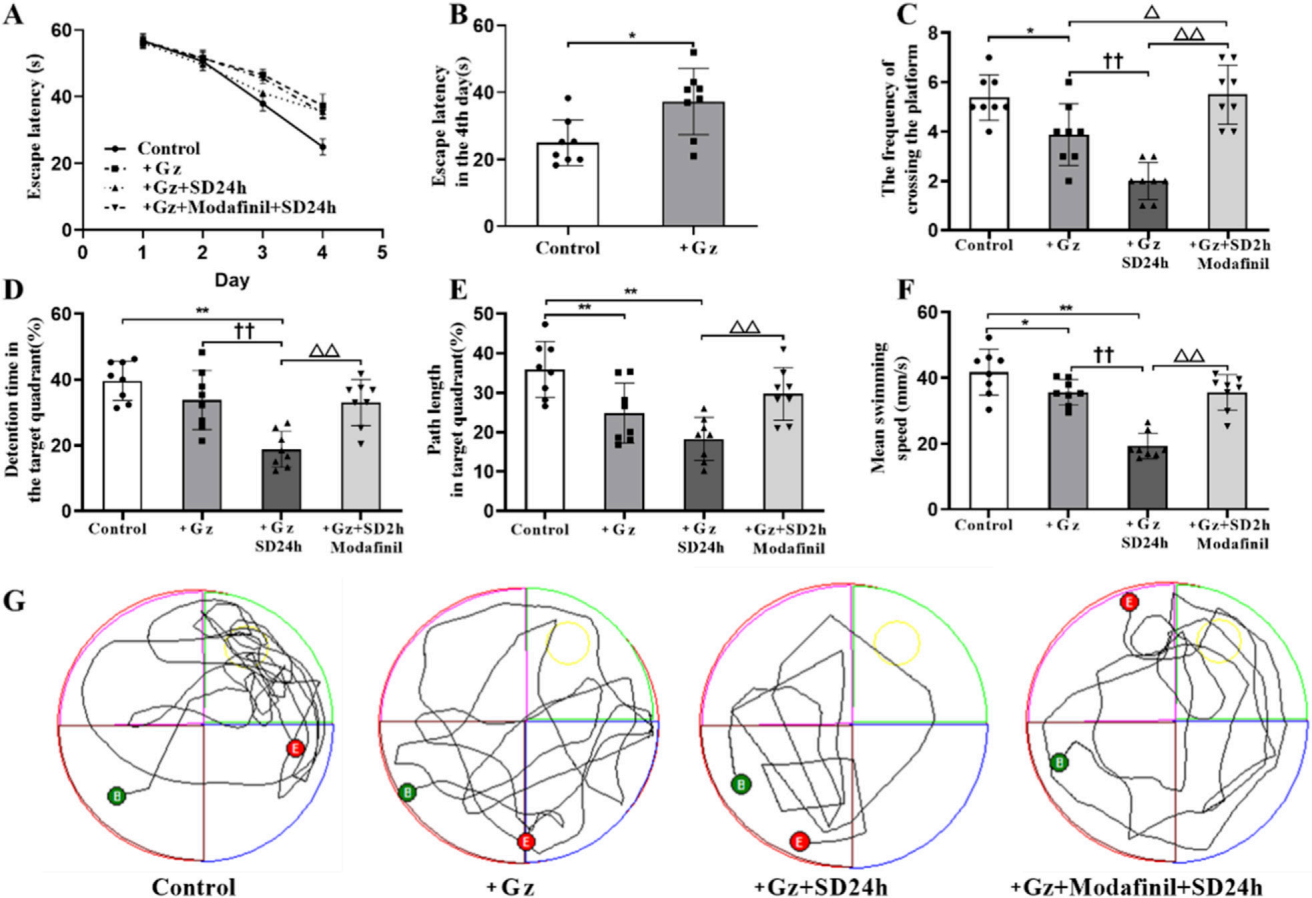
The effect of modafinil on the learning and memory abilities of mice subjected to 24-h sleep deprivation under +Gz exposure conditions. **(A)** The escape latency of mice during the first 4 days of positioning and navigation training; **(B)** The escape latency of mice in the +Gz group and the Control group on the 4th training day; **(C)** The number of times mice crossed the platform in the spatial exploration experiment; **(D)** The proportion of time spent in the target quadrant; **(E)** The proportion of swimming distance in the target quadrant; **(F)** The average swimming speed in the target quadrant; **(G)** The movement trajectory diagram of mice in the spatial exploration experiment. *P < 0.05, **P < 0.01, vs. the Control group; ^△^ *P* < 0.05, ^△△^ *P* < 0.01, vs. (+Gz + Modafinil + SD24 h) group; ^††^ *P* < 0.01 (+Gz) group vs (+Gz + SD24 h) group, n = 8.

The results demonstrated (Figures 1A,B) that throughout the first 4 days of acquisition training, the escape latency of all groups gradually decreased with repeated training sessions. However, mice in the +Gz group exhibited significantly prolonged escape latencies compared to those in the Control group (*P* < 0.05). These findings indicate that +Gz stimulation impairs spatial learning and memory performance in mice.

#### 3.1.2 Modafinil can improve the learning and memory abilities of mice under +Gz exposure and sleep deprivation conditions

In the spatial probe trial, cognitive performance was assessed by quantifying platform crossings, the percentage of time spent in the target quadrant (quadrant IV), the percentage of swimming distance within the target quadrant, and the average swimming speed in that quadrant. The results (Figures 1C–G) demonstrated that, compared with the control group, mice in the +Gz group exhibited a significant reduction in the number of platform crossings (*P* < 0.05), a decreased percentage of swimming distance in the target quadrant (*P* < 0.01), and a lower average swimming speed in quadrant IV (*P* < 0.05). These cognitive deficits were further aggravated in mice subjected to +Gz combined with 24-h sleep deprivation (+Gz + SD24 h group). Notably, administration of modafinil in the +Gz + Modafinil + SD24 h group significantly counteracted these impairments relative to the +Gz + SD24 h group. Specifically, this group showed a marked increase in the number of platform crossings (*P* < 0.01), as well as significant elevations in both the percentage of swimming distance and time spent in the target quadrant (*P* < 0.01). The average swimming speed in quadrant IV was also significantly restored (*P* < 0.01).

Collectively, these findings indicate that while +Gz exposure alone induces learning and memory deficits, the combination with 24-h sleep deprivation leads to further cognitive deterioration. In contrast, modafinil administration effectively mitigates these impairments under combined stress conditions, demonstrating its potential in ameliorating sleep deprivation–induced cognitive deficits following +Gz exposure.

### 3.2 Changes in the blood drug concentration of modafinil in mice under + Gz exposure conditions

**FIGURE 2:**
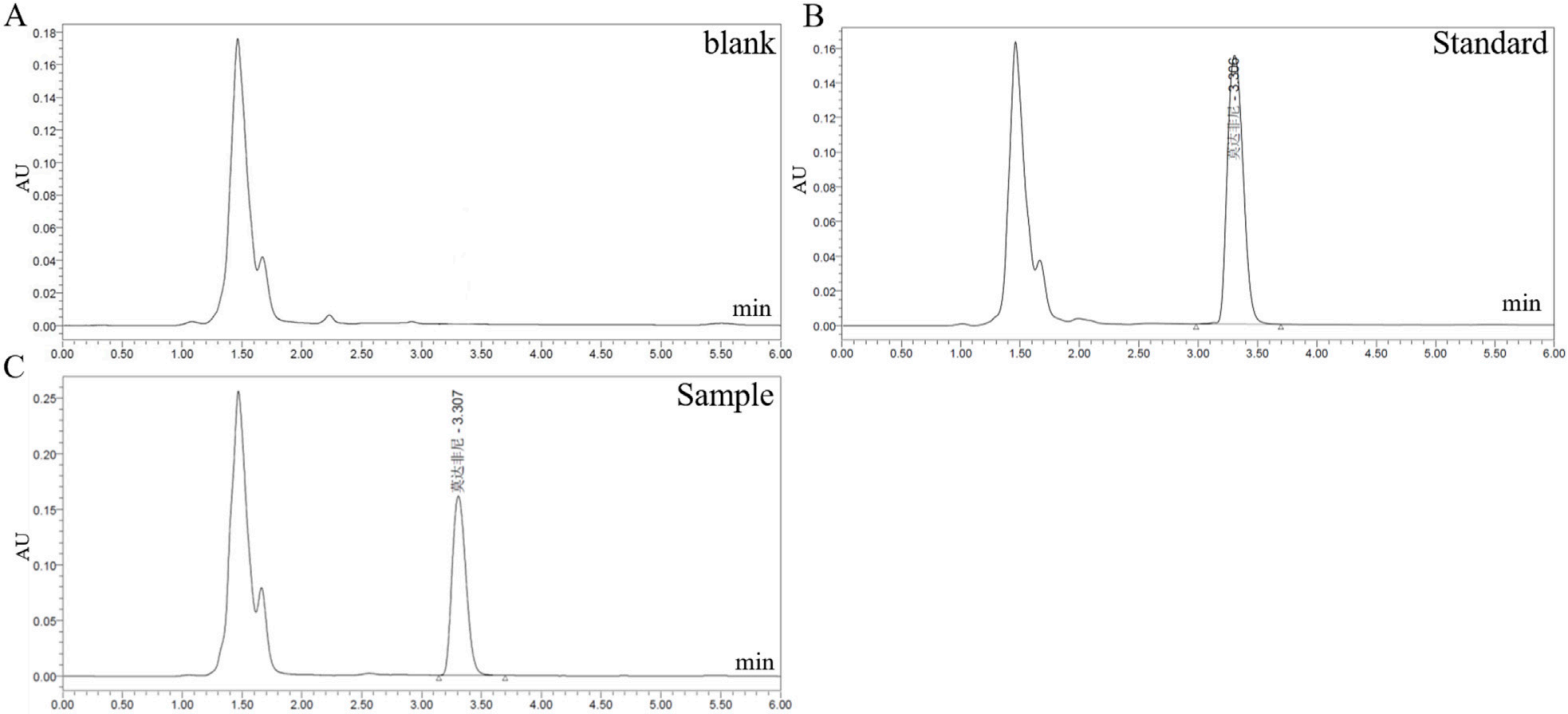
Chromatograms of modafinil (MOD) in mice plasma. **(A)** Chromatograms of blank plasma; **(B)** Chromatograms of modafinil standard; **(C)** Chromatograms of modafinil at 2 h after a single dose of 120 mg/kg in mice plasma.

The chromatograms of blank plasma did not show any peaks (Figure 2A). Figure 2B displays representative chromatograms of a modafinil standard and modafinil in mouse plasma 2 h after a single oral dose of 120 mg/kg (Figure 2C). To evaluate the specificity of the assay method, a blank plasma sample, a modafinil standard, and modafinil 12 h after oral intake were analyzed. None of the samples showed interference from endogenous substances on the retention times of modafinil, which were 3.31 min. The calibration curve for modafinil was established with equation Y = 185,03x - 4635.8, where Y represents the peak area and x indicates the concentration of modafinil. This equation exhibited a high coefficient of determination (R^2^) of 0.9998. The lower limit of quantitation (LLOQ) was identified as 0.5 mg/L, and the assay showed outstanding linearity within the concentration range of 0.5–100 mg/L (Supplementary Table S1).

The accuracy and precision of modafinil administration, both within-day and between-day, are summarized in Tables 1, 2. Variability is presented as relative standard deviation (RSD%), while accuracy is shown as a percentage; within-day and between-day precision values were ≤8.67%, and accuracy ranged from 100.7% to 98.7%. The average recoveries for plasma samples at low, medium, and high concentrations were 98.6%, 98.1%, and 99.1%, respectively (see Supplementary Tables S2, 3). To assess stability, four samples at low (1 mg/L), medium (10 mg/L), and high (50 mg/L) concentrations were prepared and evaluated under various conditions: at room temperature for 4 h, at 4 °C for 24 h, at −20 °C for 10 days, and at −80 °C for 30 days. The murine plasma samples demonstrated stability for 4 h at room temperature. Additionally, no significant degradation was observed during freeze-thaw cycles, and the plasma samples remained stable for at least 30 days at −80 °C (see Supplementary Table S4).

**TABLE 2:** Pharmacokinetic profile of modafinil in mice (‾x ± s).

Parameter	Control group	+Gz group
AUC_0-t_ (mg·h/L)	92.422 ± 13.941	88.859 ± 11.930
${\text{AUC}}_{0-\infty }$ (mg·h/L)	94.252 ± 14.622	94.467 ± 11.843
MRT_0-t_(h)	2.188 ± 0.089	2.450 ± 0.092^**^
${\text{MRT}}_{0-\infty }$ (h)	2.246 ± 0.081	2.717 ± 0.127^**^
*t*_1/2z_ (h)	1.298 ± 0.395	1.899 ± 0.203^**^
T_max_ (h)	0.5	0.5
Vz/F (L/kg)	2.427 ± 0.775	3.538 ± 0.664^*^
CLz/F (L/h/kg)	1.301 ± 0.217	1.287 ± 0.163
C_max_ (mg/L)	44.289 ± 7.372	34.182 ± 8.158^*^

Abbreviations**:** AUC, area under the plasma concentration-time curve; MRT, mean residence time; t_1/2z_, elimination half-life; T_max_, time to reach C_max_; Vz/F, apparent volume of distribution; CLz/F, apparent clearance; C_max_, peak plasma concentration.

Data are expressed as mean ± SD. *P < 0.05 and **P < 0.01 compared with the control group, n = 6 per time point, total 40–50 mice per group.

**FIGURE 3:**
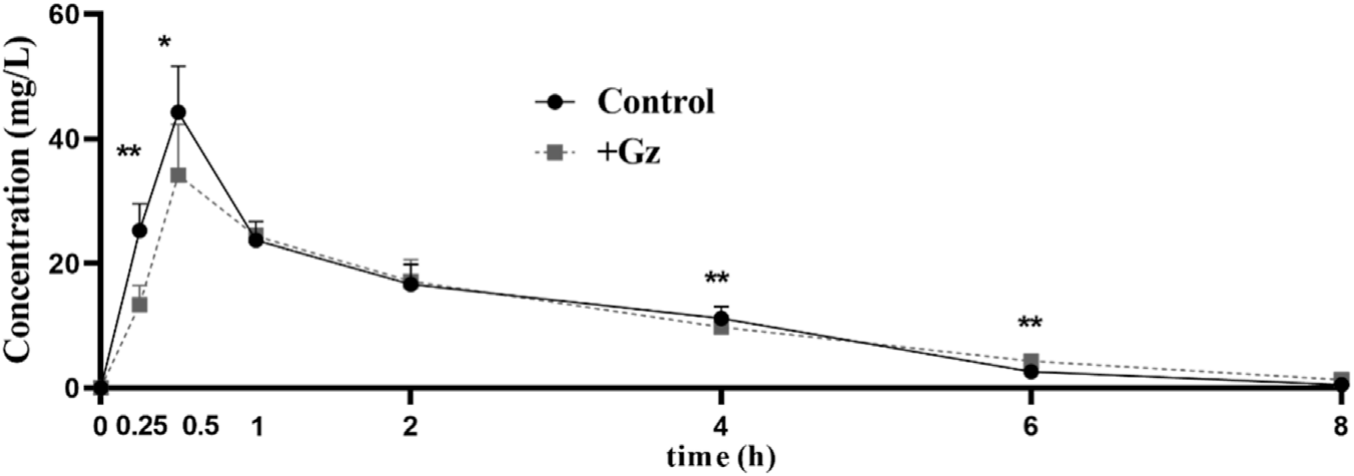
The blood concentration-time curves of modafinil drugs in the +Gz exposure group and the control group. *P < 0.05, **P < 0.01, vs. the control group, n = 40–50.

Adult male C57BL/6J mice were administered modafinil (120 mg/kg) following daily +Gz exposure over 7 consecutive days. To characterize the pharmacokinetic profile, serial blood samples were collected via the orbital fossa at 15 and 30 min, and 1, 2, 4, 6, and 8 h post-administration, with subsequent determination of plasma modafinil concentrations. The results (Figure 3) demonstrated a significant alteration in the pharmacokinetics of modafinil induced by +Gz exposure. Specifically, during the initial absorption and distribution phase, the plasma concentration of modafinil in the +Gz group was substantially lower than that in the control group at the 15-and 30-min time points (*P* < 0.01 and *P* < 0.05, respectively). Conversely, during the later clearance phase, the +Gz group exhibited higher drug concentrations compared to the control group at 4 and 6 h (*P* < 0.01).

### 3.3 Pharmacokinetic parameters of modafinil in mice

The plasma concentration-time profile of modafinil following intragastric administration in mice was analyzed using the pharmacokinetic software DAS 2.0. The data were best described by a two-compartment model. Key pharmacokinetic parameters are summarized in Table 2. Comparative analysis revealed significant alterations in the +Gz group relative to the control group. Notably, the mean residence time (MRT_0–t_ and ${\text{MRT}}_{0–\infty }$ ) was significantly prolonged (*P* < 0.01 for both), and the elimination half-life (t_1/2z_) was markedly increased (*P* < 0.01). The apparent volume of distribution (Vz/F) was also significantly larger (*P* < 0.05), while the peak drug concentration (C_max_) was significantly reduced (*P* < 0.05). In contrast, no statistically significant differences were observed between the two groups in the area under the plasma concentration-time curve (AUC_0–t_, ${\text{AUC}}_{0–\infty }$ ), clearance (CLz/F), or time to peak concentration (T_max_) (*P* > 0.05 for all).

### 3.4 Changes in liver function of mice administered modafinil under +Gz exposure conditions

**FIGURE 4:**
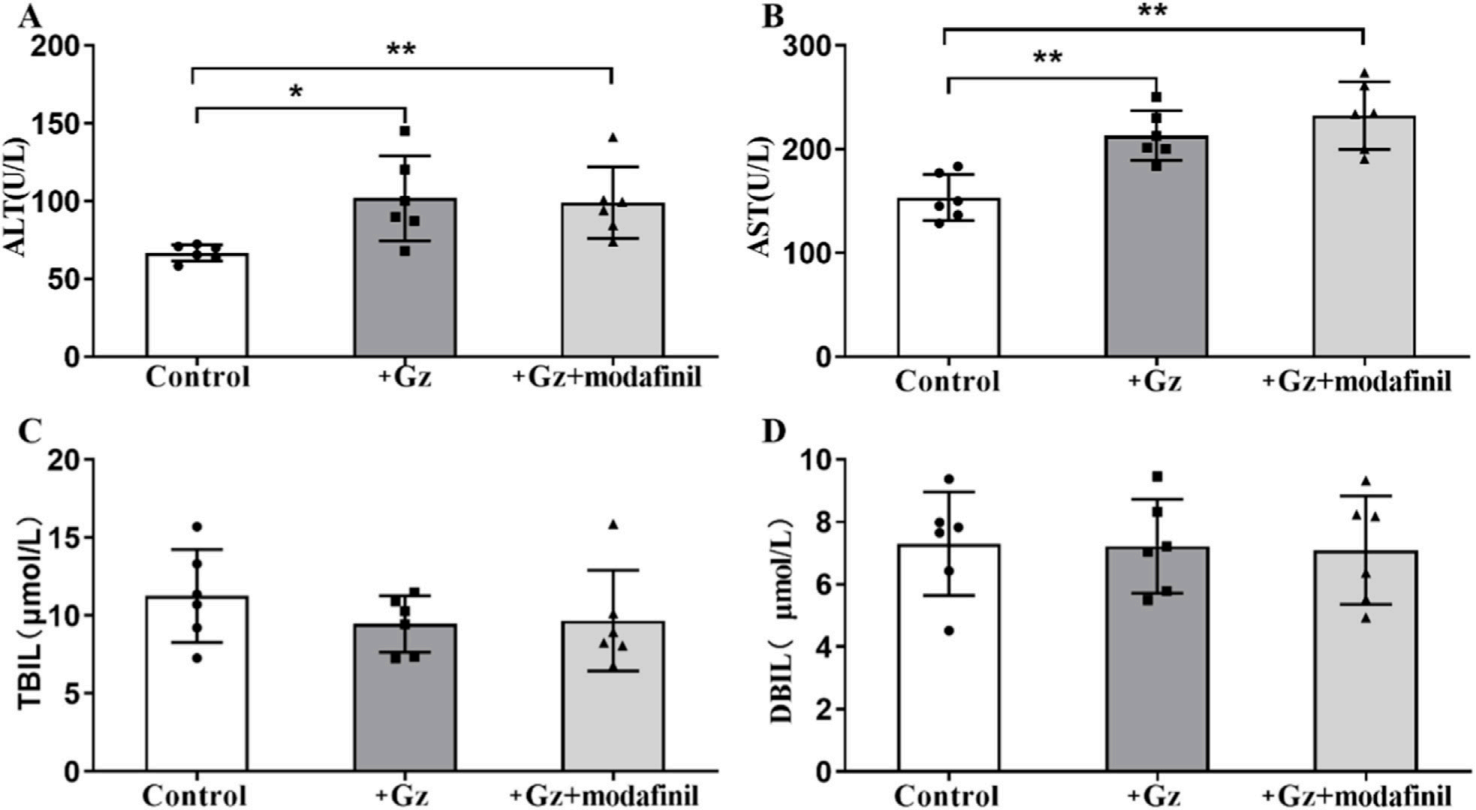
The effect of modafinil on liver function in mice exposed to +Gz. **(A)** Alanine aminotransferase; **(B)** Aspartate aminotransferase; **(C)** Total bilirubin; **(D)** Direct bilirubin. *P < 0.05, **P < 0.01, vs. Control group, n = 6.

C57BL/6J male adult mice were subjected to a +Gz intervention (15 G, 15 min per day) for 7 consecutive days. Following this exposure, modafinil was administered via gavage to evaluate its effect on liver function under +Gz conditions. The results, as shown in Figure 3, indicated that compared with the control group, the mice in the +Gz exposure group and the modafinil combined with +Gz group exhibited a slight increase in ALT and AST levels (*P* < 0.05, *P* < 0.01, respectively). However, the levels of TBIL and DBIL in both of these groups did not show a significant difference from those in the control group (*P* > 0.05) (Figure 4).

### 3.5 Gene Ontology enrichment analysis of differentially expressed genes in the liver of mice administered modafinil under + Gz exposure

**FIGURE 5:**
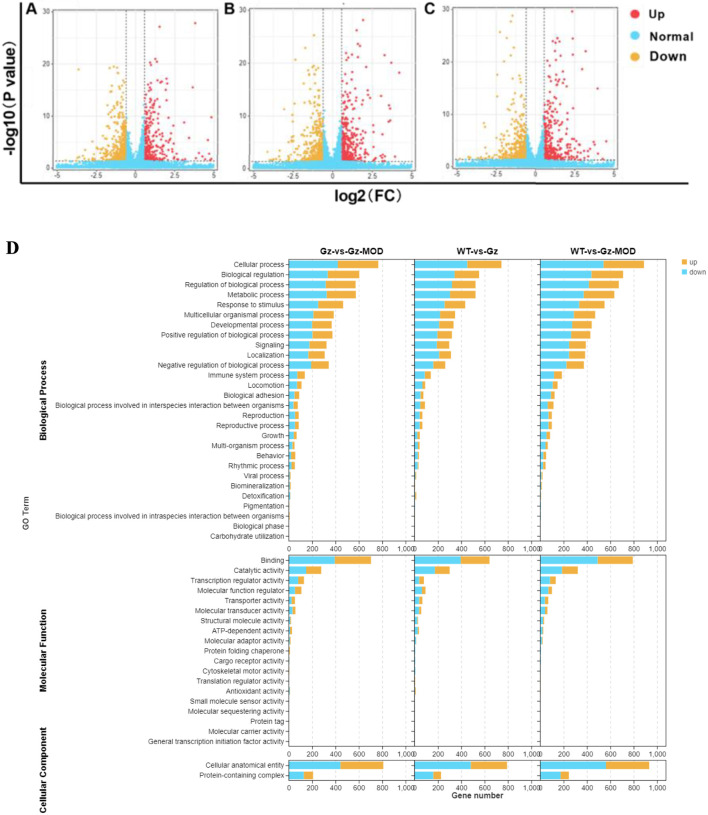
Gene Ontology Enrichment Analysis of Differentially Expressed Genes in the Liver of Mice Administered Modafinil under +Gz Exposure. **(A)** Differential gene expression compared between +Gz group and control group; **(B)** Differential gene expression compared between +Gz combined with modafinil group and +Gz group; **(C)** Differential gene expression compared between +Gz combined with modafinil group and control group. **(D)** Functional annotation of differentially expressed genes via gene ontology (GO) enrichment analysis.

Transcriptomic analysis of mouse liver tissues from the control, +Gz, and +Gz combined with modafinil groups was carried out to assess the effects of +Gz and modafinil on the expression of drug metabolism-related genes. The DEseq2 software was employed to analyze inter-group differential gene expression, and the results were quantified and visualized (Figures 5A–C). The findings indicated that, relative to the control group, the +Gz group had 179 differentially expressed genes (83 upregulated, 96 downregulated). In contrast, the +Gz combined with modafinil group, when compared to the +Gz group, contained 228 differentially expressed genes (114 upregulated, 114 downregulated). Direct comparison between the +Gz combined with modafinil group and the control group identified a total of 230 differentially expressed genes, consisting of 132 upregulated and 98 downregulated genes.

To elucidate the functional implications of hepatic differentially expressed genes (DEGs) in mice treated with modafinil under +Gz exposure, we conducted Gene Ontology (GO) enrichment analysis, which systematically annotated DEGs into three functional categories: biological process (BP), molecular function (MF), and cellular component (CC) (Figure 5D). In the BP category, DEGs were significantly enriched in oxidative stress response, lipid metabolic process, and cellular detoxification process—biological events tightly linked to +Gz-induced hepatic stress and the regulatory effects of modafinil. Notably, the oxidative stress response term was characterized by a predominance of upregulated DEGs, indicative of an enhanced hepatic antioxidant defense system triggered by the combined stimuli. For MF, the most prominently enriched terms included protein binding, hydrolase activity, and growth factor activity; among these, hydrolase activity (a key mediator of metabolic regulation) exhibited a mixed pattern of up- and downregulated DEGs, reflecting the complex metabolic remodeling in the liver under modafinil treatment and +Gz exposure. In the CC category, DEGs were concentrated in cell membrane, mitochondrial matrix, and cytosolic vesicle, with membrane-associated DEGs mostly upregulated, suggesting that modafinil may modulate hepatic cellular transport and mitochondrial energy metabolism under +Gz exposure.

### 3.6 Analysis of KEGG enrichment pathways for differentially expressed genes in the liver of mice administered modafinil under +Gz exposure conditions

**FIGURE 6:**
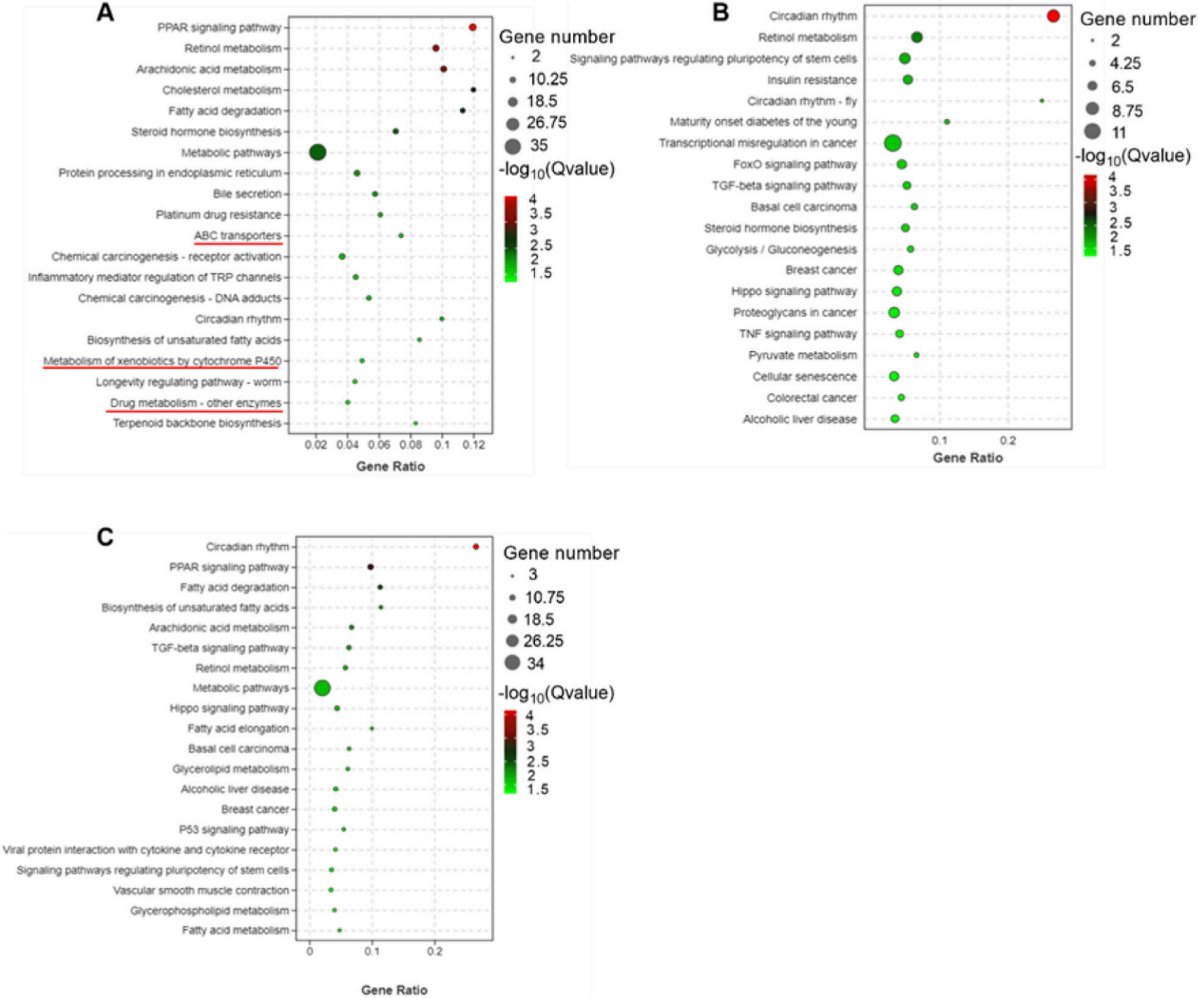
KEGG enrichment analysis of differentially expressed genes in the liver of mice administered modafinil under +Gz exposure conditions. **(A)** KEGG enrichment analysis results of the +Gz group and the control group; **(B)** KEGG enrichment analysis results of the +Gz combined with modafinil group and the +Gz group; **(C)** KEGG enrichment analysis results of the +Gz combined with modafinil group and the control group. The figure shows the top 20 pathways of differentially expressed genes.

The KEGG database integrates genomic, biomolecular, and systemic functional information, allowing for the mapping of differentially expressed genes (DEGs) identified from sequencing to specific biological systems, including organism-level, cellular, and signaling pathway functions. It encompasses eight major analytical categories, such as human diseases and drug development. By comparing our sequencing-derived DEGs against the KEGG database, we obtained the following functional enrichment results (Figure 6). Notably, compared to the control group, DEGs in the livers of the +Gz group were significantly enriched in multiple drug metabolism signaling pathways, including cytochrome P450-mediated drug metabolism, metabolism by other enzymes, and ABC transporters. In contrast, when comparing the +Gz combined with modafinil group to the +Gz group, the DEGs were significantly enriched in pathways related to glycolipid metabolism, transcriptional regulation, as well as FOXO, TGF-β, and TNF signaling pathways. Furthermore, relative to the control group, the DEGs in the +Gz combined with modafinil group showed significant enrichment in various metabolic pathways, such as fatty acid metabolism, transcriptional regulation, cholesterol and arachidonic acid metabolism, alongside multiple signaling pathways including PPAR and TGF-β.

### 3.7 Clustering analysis of differentially expressed genes in the liver of mice administered modafinil under +Gz exposure conditions

**FIGURE 7:**
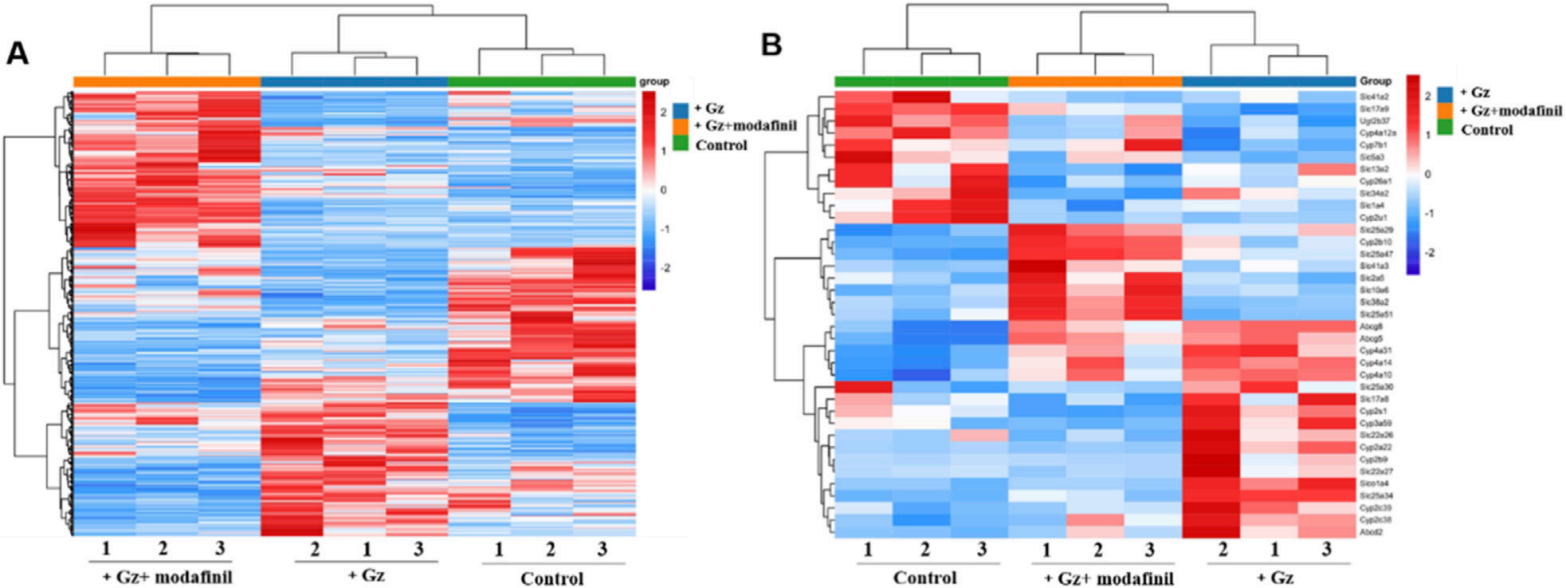
Clustering analysis of differentially expressed genes in the liver of mice administered modafinil under +Gz exposure conditions. **(A)** Clustering analysis of all DEGs in the three groups; **(B)** Clustering analysis of drug metabolism-related differentially expressed genes.

Based on the standardized average FPKM values of differentially expressed genes (DEGs) among the control, +Gz, and +Gz combined with modafinil groups, we first performed cluster analysis on all DEGs. The results (Figure 7A) indicated a substantial number of DEGs across the three groups. Building on this, we further screened genes closely associated with drug absorption, distribution, metabolism, and excretion (ADME) for hierarchical clustering analysis. The results (Figure 7B) revealed a total of 37 ADME-related DEGs among the three groups.

Specifically, compared with the control group, the +Gz group showed downregulation of drug metabolism-related genes such as *Slc17a9, Slc41a2, Ugt2b37, Cyp2u1, and Cyp4a12a*, while genes including *Slc25a34, Abcd2, Abcg5, Abcg8, Cyp2c39*, and *Cyp2c38* were upregulated. In the +Gz combined with modafinil group, compared with the +Gz group, genes such as *Slc25a29, Slc25a47*, and *Cyp2b10* were significantly upregulated, whereas *Slc25a34, Abcd2, Abcg5, Abcg8, Cyp2c39*, and *Cyp2c38* were downregulated. Furthermore, relative to the control group, the +Gz combined with modafinil group exhibited significant upregulation of *Slc25a29, Slc25a47*, and *Cyp2b10*, and downregulation of *Slc17a9, Slc41a2, Ugt2b37, Cyp2u1*, and *Cyp4a12a*.

### 3.8 Verification of the expression of liver drug metabolism-related DEGs in mice administered modafinil under +Gz exposure conditions

**FIGURE 8:**
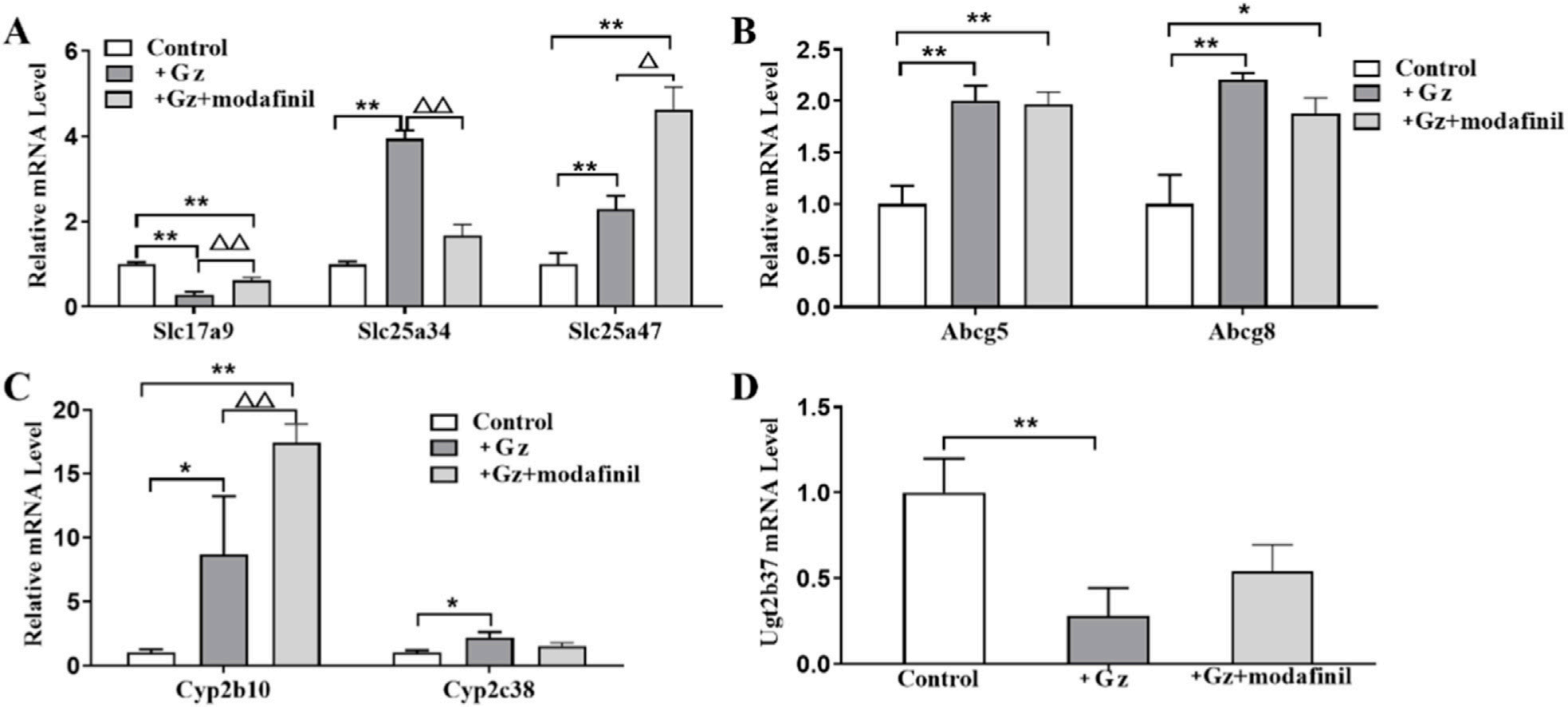
Validation of the expression of liver drug metabolism-related DEGs in mice administered modafinil under +Gz exposure conditions. **(A)** mRNA expression of genes related to liver drug absorption (Slc17a9, Slc25a34, Slc25a47); **(B)** mRNA expression of genes related to liver drug excretion (Abcg5, Abcg8); **(C)** mRNA expression of genes related to liver drug metabolism (Cyp2b10, Cyp2c38); **(D)** mRNA expression of Ugt2b37 gene. *P < 0.05, **P < 0.01, vs. control group; △*P* < 0.05, △△*P* < 0.01, vs. +Gz group, n = 3.

The transcriptomic sequencing results described above indicate significant alterations in hepatic drug metabolism-related genes in mice treated with modafinil under +Gz exposure. To validate these findings, we selected eight genes (*Abcg5, Abcg8, Ugt2b37, Slc17a9*, *Slc25a47, Slc25a34, Cyp2b10*, and *Cyp2c38*) that were markedly affected by +Gz and are closely implicated in drug metabolism for qPCR validation. The mRNA expression levels of these genes were determined by RT-qPCR. As shown in Figure 8, compared with the control group, the +Gz group exhibited downregulated expression of *Slc17a9* and *Ugt2b37*, while the expression of *Slc25a34, Slc25a47, Abcg5, Abcg8, Cyp2b10*, and *Cyp2c38* was upregulated. In the +Gz combined with modafinil group, the expression of *Slc17a9* was downregulated, whereas *Slc25a47, Abcg5, Abcg8*, and *Cyp2b10* were upregulated relative to the control. Furthermore, a direct comparison between the +Gz combined with modafinil group and the +Gz group revealed upregulated expression of *Slc17a9*, *Slc25a47*, and *Cyp2b10*, but downregulated expression of *Slc25a34*; the other genes showed no statistically significant differences.

### 3.9 Expression and activity of the liver drug-metabolizing enzyme Cyp3a11 in mice administered modafinil under +Gz exposure conditions

**FIGURE 9:**
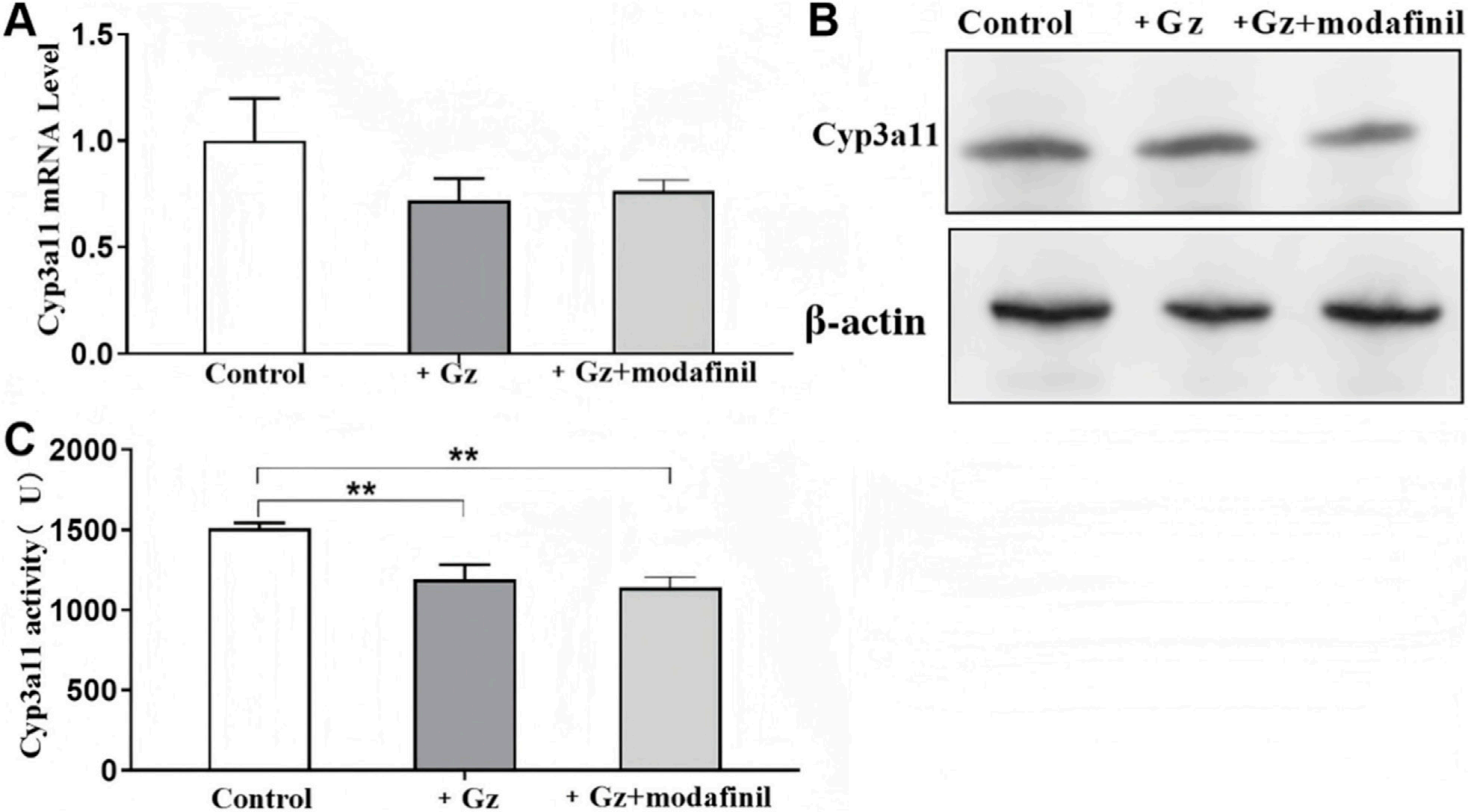
Changes in the expression and activity of Cyp3a11 in the liver of mice administered modafinil under +Gz exposure conditions. **(A)** mRNA expression of Cyp3a11 in the liver of mice; **(B)** protein expression of Cyp3a11 in the liver of mice; **(C)** The activity of Cyp3a11 enzyme in the liver of mice. *P < 0.05, **P < 0.01, n = 6.

Transcriptome sequencing analysis revealed no significant alterations in the mRNA expression of *Cyp3a11*, a principal hepatic drug-metabolizing enzyme for modafinil, under +Gz exposure. To further investigate the potential effects of acceleration, we re-evaluated Cyp3a11 expression using RT-qPCR and Western blot analysis (Figures 9A,B). The results were consistent with the transcriptomic data, demonstrating no significant changes in either the mRNA level or the corresponding protein abundance of Cyp3a11 in mouse liver following +Gz exposure (*P* > 0.05). Notwithstanding these findings, subsequent assessment of Cyp3a11 enzymatic activity via an ELISA kit revealed a significant reduction. As shown in Figure 9C, the Cyp3a11 activity in both the +Gz group and the +Gz combined with modafinil group was markedly lower than that in the Control group (*P* < 0.01 for both comparisons).

## 4 Disscusion

Repetitive high +Gz acceleration, a pivotal environmental stressor in military aviation and aerospace missions, disrupts hemodynamic balance, tissue oxygenation, and systemic homeostasis, posing comprehensive physiological challenges to multiple organ systems (Soh et al., 2024; Öztürk et al., 2012). Consistent with prior preclinical research, our findings confirm that sustained +15 Gz exposure (7 days, 15 min/day) induces subclinical hepatic injury, characterized by elevated serum ALT and AST-hallmarks of hepatocellular damage—in the absence of bilirubin elevation. This pattern aligns with Shi et al.‘s observation that +Gz stress triggers hepatic oxidative stress and structural disarray (Shi et al., 2019), and resonates with the “adaptive-damaging” dual response to chronic mechanical stress proposed by Li et al. (Shi et al., 2015; Lemaigre, 2019). Importantly, our study extends these insights by linking +Gz-induced liver injury to specific perturbations in drug metabolism pathways, revealing functional impacts on drug-biotransforming enzymes-a critical advance for aerospace pharmacology. While previous studies focused predominantly on +Gz-induced cardiovascular or central nervous system effects, our work positions the liver as a key pharmacotherapeutic target, particularly relevant for aviators who rely on medications to maintain operational readiness during and after high-G maneuvers.

Hepatic drug metabolism is orchestrated by a complex network of cytochrome P450 (CYP) enzymes, membrane transporters, and phase II conjugation systems (Rodrigues et al., 2022; Chen et al., 2018; Li et al., 2021). Our transcriptomic analysis revealed that +Gz exposure systematically reprograms this network: we observed downregulation of uptake transporters such as Slc17a9, upregulation of efflux transporters including Abcg5/Abcg8 and Slc25a family members, and altered expression of CYP isoforms (Cyp2b10, Cyp2c38) and conjugation enzymes (Ugt2b37). These changes are consistent with general stress-induced metabolic adaptations, but the specific pattern we observed-particularly the coordinated upregulation of multiple efflux transporters alongside suppressed uptake capacity-suggests a shift toward enhanced drug elimination at the transport level. This transcriptional reprogramming may have broad implications for aerospace pharmacotherapy: melatonin clearance could be impaired due to Ugt2b37 downregulation (Mokkawes and de Visser, 2023), while anti-inflammatory agents such as ibuprofen and naproxen may exhibit accelerated clearance via Cyp2c38 upregulation (Oliveira et al., 2022; Nair et al., 2023). Furthermore, drugs with narrow therapeutic windows-including loratadine, propofol, and warfarin—may display unpredictable pharmacokinetics under + Gz stress (Zhang et al., 2021), highlighting the pressing need for a “Gz-drug interaction” framework in aeromedical pharmacology. Recent studies have successfully employed multi-omics integration-combining 16 S rRNA sequencing, metabolomics, network pharmacology, and transcriptomics—to elucidate the complex mechanisms of liver diseases such as MAFLD (Jia et al., 2026), demonstrating the power of such integrative approaches in dissecting hepatic metabolic regulation under pathological conditions. Applying similar multi-layered analytical strategies to aerospace pharmacology may further unravel the intricate network of drug metabolism under +Gz stress.

Among these transcriptomic alterations, the most striking and mechanistically revealing finding concerns Cyp3a11, the principal murine enzyme responsible for modafinil metabolism (orthologous to human CYP3A4). Our data from transcriptomic sequencing, RT-qPCR, and Western blotting consistently demonstrated that +Gz exposure did not alter Cyp3a11 mRNA or protein levels. In parallel, however, enzymatic activity assays performed under identical conditions revealed a significant suppression of Cyp3a11 catalytic function. It challenges the conventional assumption that changes in drug-metabolizing enzyme activity are invariably accompanied by corresponding changes in transcript or protein abundance, and instead points toward previously unrecognized regulatory layers operative under unique physiological stressors. Mechanistically, this phenomenon raises several testable hypotheses. Post-translational modifications-including oxidative carbonylation (given the well-documented +Gz-induced oxidative stress), phosphorylation, or S-nitrosylation-could alter the enzyme’s active-site conformation or substrate-binding affinity without affecting its immunoreactive protein levels (Lin et al., 2012; Tripathi et al., 2017). Alternatively, reduced heme availability or incorporation into the apoprotein, as well as alterations in the membrane lipid microenvironment that affect CYP-reductase interactions, may account for the functional suppression. Competitive inhibition by endogenously accumulated metabolites under stress conditions represents another plausible mechanism. These possibilities are not mutually exclusive and may operate in concert. Notably, this pattern contrasts sharply with prior reports of stress-induced CYP3A transcriptional downregulation under hypoxia or chemical stressors (Andrade, 2023), underscoring that +Gz exerts a unique post-translational regulatory influence on CYP enzymes-a mechanism rarely reported in aerospace medicine.

An additional regulatory layer that warrants consideration is the hepatic peripheral circadian clock. The liver possesses a robust, cell-autonomous peripheral oscillator in which core clock genes (Bmal1, Clock, Per1/2, Cry1/2) form transcriptional-translational feedback loops driving rhythmic expression of approximately 10% of the hepatic transcriptome (Hunter and Ray, 2019). Importantly, multiple CYP450 family members-including Cyp3a11-are under direct or indirect clock control. Bmal1 ablation markedly blunts the circadian rhythms of both Cyp3a11 expression and its enzymatic activity (Lin et al., 2019); clock output mediators DBP and DEC2 respectively activate and repress Cyp3a11 transcription (Zhang et al., 2018). Thus, any factor that disrupts hepatic clock rhythmicity could potentially alter Cyp3a11 catalytic function-without changing its gene copy number-by affecting its post-translational modifications or cofactor availability. Stress is a well-established modulator of peripheral clock rhythmicity: social defeat stress and glucocorticoids significantly alter PERIOD2 rhythmicity in the liver while the central clock (SCN) remains resistant (Ota et al., 2020), indicating that peripheral clocks are highly sensitive to stress signals. In the present study, repeated + Gz exposure-a potent mechanical and oxidative stressor-may disrupt hepatic clock function through hemodynamic fluctuations, oxidative stress, and glucocorticoid signaling, thereby indirectly suppressing Cyp3a11 activity via clock-dependent post-translational pathways. This hypothesis, though requiring direct experimental validation, offers a novel theoretical framework linking mechanical stress, circadian biology, and drug metabolism-a nexus previously unexplored in aerospace medicine.

The functional consequence of these molecular alterations is most clearly manifested in the pharmacokinetics of modafinil. Our behavioral data confirmed that modafinil retains its cognitive-protective efficacy under +Gz stress: it significantly reversed the learning and memory deficits induced by + Gz exposure alone and in combination with 24-h sleep deprivation, validating its continued utility in high-G environments. However, pharmacokinetic analysis revealed a distinct “delayed absorption-impaired clearance” profile in +Gz-exposed mice: a 22.8% reduction in C_max_, a 12.0% prolongation of MRT_0-t_, and a 46.4% extension of t_1/2z_. Notably, T_max_ remained unchanged and AUC was preserved, indicating that the absorption rate (Ka) was reduced rather than absorption extent (bioavailability). This pattern is pharmacokinetically coherent with +Gz-induced hemodynamic redistribution-reduced splanchnic perfusion during the absorption phase impairs the rate of drug entry into the systemic circulation, while expanded volume of distribution (Vz/F increased ∼46%) prolongs the residence time without compromising net clearance (CLz/F unchanged). Importantly, the preserved CLz/F, coupled with suppressed Cyp3a11 activity, suggests that the enzyme’s intrinsic metabolic capacity may be maintained at baseline but is functionally constrained under stress-perhaps due to substrate access limitations or cofactor insufficiency. This integrated interpretation reconciles the seemingly contradictory PK parameters and provides a mechanistic linkage from molecular enzyme suppression to whole-organism drug disposition.

From a clinical and operational perspective, these findings carry implications for aerospace pharmacotherapy. The prolonged elimination half-life and extended mean residence time of modafinil under +Gz conditions suggest that standard dosing intervals may lead to drug accumulation during repeated administration over multi-day missions. We propose that a 30%–50% extension of the dosing interval should be considered for prolonged high-G operations to avoid potential adverse effects while maintaining therapeutic efficacy (Chiniard et al., 2023). Moreover, the differential regulation of various CYP isoforms and transporters observed in our transcriptomic data implies that other medications commonly used in aviation-including analgesics, antihistamines, and sedatives-may exhibit altered pharmacokinetics under similar stress conditions, necessitating individualized dose adjustments and careful monitoring.

Despite these contributions, several limitations warrant acknowledgment. First, we utilized a murine model, and interspecies differences in cytochrome P450 profiles-particularly the functional divergence between murine Cyp3a11 and human CYP3A4-as well as varying physiological responses to +Gz stress, may hinder direct translational extrapolation to pilots and astronauts. Second, our +Gz exposure protocol (15 min daily for 7 consecutive days) reflects a short-term repeated stress paradigm, whereas aviators often experience intermittent high-G exposure over months or even years; the long-term effects of sustained + Gz stress on drug metabolic dynamics remain unclear. Third, our investigation focused exclusively on modafinil and a limited subset of drug metabolism-related genes; broader screening across a wider range of pharmaceuticals and metabolic pathways is needed. Fourth, the mechanistic interpretation of Cyp3a11 suppression-whether via oxidative modification, peripheral clock disruption, or other pathways-remains hypothetical and requires direct experimental validation through targeted proteomics, activity-based probes, and clock gene perturbation studies. Finally, the fixed administration time in our study limits our ability to assess chronopharmacological influences; future studies comparing different dosing times (ZT0 vs. ZT12) would help clarify the role of circadian rhythms in the observed effects (Tani et al., 2023).

In conclusion, intermittent high-G exposure reprograms hepatic drug metabolism and functionally inhibits Cyp3a11, altering the pharmacokinetics of modafinil while preserving its efficacy in +Gz exposure environments.

## Data Availability

The original contributions presented in the study are included in the article/Supplementary Material, further inquiries can be directed to the corresponding authors.
