# Supplementary material for: Alterations in the hepatic drug-metabolizing enzyme network of mice induced by +Gz exposure and its impact on the pharmacokinetics and pharmacodynamics of modafinil
Source: Front Pharmacol. 2026 Aug 3;17:1818737. doi: 10.3389/fphar.2026.1818737 (PMC13478242; doi:10.3389/fphar.2026.1818737)
Supplement: Supplementary file 1 [file DataSheet1.pdf]

Supplemental Tables

Supplemental Table 1. Regression equation, linear range, correlation coefficient, and LLOQ of modafinil in mouse plasma

Compound	Standard curve regression equation	Linear range (mg/L)	R^2	LLOQ (mg/L)
Modafinil	$Y = 18503\chi - 4635.8$	0.5 ~ 100	0.9998	0.5

Supplemental Table 2. Assessment of within-day accuracy, precision（$\overline{X}$±s, n=6）

Actual concentration (mg/L)	Measured concentration (mg/L)	Accuracy	Precision
1	1.007 ± 0.070	1.007	0.070
10	10.020 ± 0.339	1.002	0.034
50	49.370 ± 1.308	0.987	0.026

Supplemental Table 3. Assessment of between-day accuracy, precision（$\overline{X}$±s, n=6）

Actual concentration (mg/L)	Measured concentration (mg/L)	Accuracy	Precision
1	1.007 ± 0.087	1.007	0.087
10	9.967 ± 0.444	0.997	0.044
50	49.370 ± 1.586	0.987	0.032

Supplemental Table 4. Assessment of stability（$\overline{X}$±s, n=6）

Storage conditions	Actual concentration(mg/L)	Measured concentration(mg/L)	RSD(%)
Room temperature (4 h)	1	1.036 ± 0.082	7.918
	10	10.260 ± 0.504	4.910
	50	49.110 ± 1.607	3.272
4°C (24 h)	1	0.980 ± 0.050	5.063
	10	9.766 ± 0.666	6.817
	50	49.640 ± 2.349	4.732
-20°C (10 days)	1	0.988 ± 0.059	5.954
	10	9.820 ± 0.447	4.549
	50	50.190 ± 2.004	3.993
-80°C (30 days)	1	0.976 ± 0.050	5.085
	10	10.250 ± 0.464	4.528
	50	50.140 ± 1.933	3.855
